# Application and evidence gaps of sarcopenia diagnostic criteria in patients with heart failure: a scoping review

**DOI:** 10.3389/fmed.2026.1957639

**Published:** 2026-09-29

**Authors:** Mingzhe Li, Wei Lv, Ting Li, Lijun Su, Qing Zhao, Hui Xu, Jianxiu Man

**Affiliations:** Cardiovascular Disease Diagnosis and Treatment Center, Siping Central People’s Hospital, Siping, China

**Keywords:** diagnostic criteria, evidence gap, heart failure, sarcopenia, scoping review

## Abstract

**Background:**

Diagnostic criteria for sarcopenia show heterogeneity in patients with heart failure (HF). This study reviews existing evidence through a scoping review.

**Methods:**

We searched six databases (PubMed, Embase, CINAHL, Web of Science, China National Knowledge Infrastructure [CNKI], and Wanfang) from inception to May 2026. Two reviewers screened literature and extracted data independently. We used narrative synthesis.

**Results:**

We included 23 studies covering 8 diagnostic criteria. The Asian Working Group for Sarcopenia (AWGS) 2019 (9 studies) and the European Working Group on Sarcopenia in Older People 2 (EWGSOP2) (7 studies) were most common. The overall prevalence ranged from 8.9% to 65.8%, with wide variation within the same criterion. Only 3 studies performed head-to-head comparisons. Heart failure-specific factors (edema interference, New York Heart Association [NYHA] class limitation, timing of assessment in acute phase) received limited attention. No study assessed sarcopenia at acute decompensation before diuresis. One study evaluated patients with stage B HF, generally considered pre-HF, and was interpreted separately. Eleven studies reported prognostic associations, yet only 1 reported discrimination and reclassification metrics such as concordance index (C-index), net reclassification improvement (NRI), and integrated discrimination improvement (IDI). External validation of diagnostic and prognostic performance was insufficient.

**Conclusion:**

Current evidence does not support a unified heart failure-specific diagnostic framework for sarcopenia. Future work should include multicenter prospective cohort studies to establish HF-specific cut-off values, develop assessment protocols for acute decompensated HF and NYHA class IV patients, and conduct large-sample external validation of diagnostic and prognostic performance.

## 1 Introduction

Sarcopenia is a geriatric syndrome characterized by progressive and generalized loss of skeletal muscle mass and function (1). In patients with heart failure (HF), this problem carries a heavy clinical burden. A meta-analysis showed that the pooled prevalence of sarcopenia in HF patients is about 30% (2). Coexisting sarcopenia and HF worsens patient outcomes. Sarcopenia is an independent risk factor for adverse prognosis in both heart failure with reduced ejection fraction (HFrEF) and heart failure with preserved ejection fraction (HFpEF) (3). The two conditions form a pathophysiological vicious cycle (4). Early identification of sarcopenia and timely intervention may help prevent functional decline and adverse outcomes (5, 6). Sarcopenia, frailty, and cachexia are overlapping but distinct clinical entities, and available evidence does not establish a single inevitable progression from one to another (7–9). Most diagnostic studies have enrolled hospitalized patients with symptomatic HF; evidence in stage B HF, which is generally considered pre-HF, remains limited. This evidence gap limits evaluation of whether systematic assessment during earlier HF stages could improve risk stratification and guide timely intervention.

Sarcopenia and cachexia overlap biologically, but sarcopenia may be more responsive to nutritional and exercise interventions than established cachexia (7–9). Accurate diagnosis is a prerequisite for nutritional support, exercise rehabilitation, and drug therapy. Delayed or non-standardized diagnosis may delay nutritional, exercise, and pharmacological treatment and rehabilitation. Multiple international diagnostic criteria for sarcopenia exist (1, 2), including EWGSOP1 and EWGSOP2 (10, 11), AWGS 2014/2019 (12, 13), the Global Leadership Initiative on Sarcopenia (GLIS), the Foundation for the National Institutes of Health (FNIH) criteria, the Sarcopenia Definitions and Outcomes Consortium (SDOC) criteria, the International Working Group on Sarcopenia (IWGS) criteria, and predefined study-specific combinations of objective indicators (2). These criteria differ in diagnostic dimensions, measurement tools, and cut-off values. The special pathophysiological state of HF adds diagnostic difficulty. Fluid retention causes edema that interferes with bioelectrical impedance analysis (BIA) and anthropometry. Acute decompensation limits cardiac function and makes gait speed and grip strength tests hard to complete. Diuretic-induced weight fluctuation affects muscle mass assessment (14).

Current diagnostic cut-off values derive mainly from community-dwelling older adults or general geriatric inpatients (12, 13), and direct extrapolation to HF populations remains insufficiently validated (15–17). HF-related hemodynamic changes, neurohormonal activation, chronic inflammation, edema, and diuretic-induced fluid shifts may alter muscle mass and function distributions and interfere with BIA and anthropometry (14–16). Criteria also differ in diagnostic dimensions, measurement tools, reference populations, and cut-off values, creating a risk of misclassification when they are applied to HF cohorts (15, 16). In addition, few studies validate these cut-offs in HF populations or report phenotype-stratified data across HFrEF, HFpEF, and acute or stable states (16). These limitations help explain the wide variation in detection rates within the same criterion and define the central research gap addressed by this review.

Existing studies also show fragmented methodological reporting. Few describe HF-specific factors such as edema, cardiac function limitation, weight fluctuation, and timing of acute vs. stable-phase assessment (2). Moreover, whether sarcopenia diagnosed by these criteria is independently associated with HF outcomes, or adds incremental predictive value beyond traditional HF risk factors, remains uncertain (3).

This review aims to: describe the types and version distribution of sarcopenia diagnostic criteria in existing literature; clarify differences in detection rates and distribution features of AWGS, EWGSOP2, GLIS, FNIH, and other criteria across HF types, NYHA classes, and acute vs. stable phases; extract measurement tools, cut-off settings, and methodological responses to HF-specific issues including edema interference, cardiac function class limitation, weight fluctuation, and acute phase measurement timing; and evaluate the prognostic associations and incremental predictive performance of sarcopenia diagnosed by different criteria for HF patient outcomes. Through systematic mapping across these dimensions, this review identifies methodological barriers and key evidence gaps, providing a basis for future development of HF-specific sarcopenia diagnostic consensus and research priorities.

## 2 Methods

### 2.1 Study design

This study used a scoping review method following the framework of Arksey and O'Malley (18) and the Joanna Briggs Institute (JBI) guidance for scoping reviews. We reported according to the Preferred Reporting Items for Systematic Reviews and Meta-Analyses extension for Scoping Reviews (PRISMA-ScR) (19, 20). Scoping reviews suit systematic mapping of evidence in a field, identification of research gaps, and clarification of complex concepts, especially when research questions involve multiple study designs, outcome measures, and methodological heterogeneity. The study protocol was registered on the Open Science Framework (OSF) platform (registration number: https://doi.org/10.17605/OSF.IO/EYZ6W).

### 2.2 Research questions

This review built research questions around the Population-Concept-Context (PCC) framework. The questions were: What are the detection rates of different sarcopenia diagnostic criteria in HF patients, and how are they distributed across HF types, NYHA classes, and acute vs. stable phases? What measurement tools, cut-off values, and methodological responses to HF-specific issues are used when applying these criteria in HF populations? What prognostic associations and incremental predictive performance do sarcopenia diagnoses based on different criteria have for HF patient outcomes?.

### 2.3 Inclusion and exclusion criteria

Inclusion criteria followed the PCC framework. Population: patients aged 18 years or older with acute or chronic symptomatic HF, including HFrEF, HFpEF, and heart failure with mildly reduced ejection fraction (HFmrEF), or stage B HF. Stage B HF was defined as asymptomatic structural heart disease or cardiac dysfunction and was prespecified as a distinct subgroup because it is generally considered pre-HF rather than established symptomatic HF. Findings from this subgroup were interpreted separately from those obtained in patients with stages C-D HF. Concept: studies applying EWGSOP1, EWGSOP2, AWGS 2014/2019/2023, GLIS, FNIH, SDOC, IWGS, or a predefined study-specific combination of objective indicators, and reporting the measurement tools, cut-off values, and detection rates used. Context: original quantitative research articles using cross-sectional, cohort, case-control, randomized controlled trial, or quasi-experimental designs; language was restricted to Chinese or English.

Exclusion criteria were: study protocols, conference abstracts, letters, case reports with a sample size below 30, expert consensus, and guidelines; study populations not primarily composed of patients with HF; use of screening scales alone as the sole diagnostic basis, including the Strength, Assistance in walking, Rise from a chair, Climb stairs, Falls (SARC-F) questionnaire, the SARC-F combined with calf circumference (SARC-CalF) tool, and the Ishii score, without formal diagnostic criteria; failure to report specific measurement tools, cut-off values, or sarcopenia detection rates; and duplicate publications, keeping only the one with the largest sample size or most complete information.

### 2.4 Search strategy

We searched six databases—PubMed (via MEDLINE), Embase, CINAHL, Web of Science Core Collection, China National Knowledge Infrastructure (CNKI), and Wanfang—from inception to May 2026. The search strategy combined subject headings and free-text terms for heart failure, sarcopenia-related muscle outcomes, and diagnosis, screening, or prevalence. The search strategies for all six databases are provided in Supplementary Table S1. We adapted the strategy to each database and supplemented the database search by snowballing reference lists and searching Google Scholar.

### 2.5 Data extraction

We used a structured data extraction form. Two reviewers extracted data independently, cross-checked, and resolved discrepancies by consensus. Extraction covered 12 domains: basic literature information, HF population characteristics, diagnostic criterion name and version, head-to-head comparison, muscle mass/strength/function measurement tools and parameters, cut-off values, overall and stratified detection rates, measurement timing, discussion of HF-specific issues (edema interference, cardiac function limitation, weight fluctuation, acute phase timing), test completion rates and reasons for failure, prognostic validation data (follow-up duration, outcome type, effect size, predictive performance metrics), study limitations and author discussion, and evidence gap markers. Missing data were marked as NR (not reported); non-applicable items were marked as NA. Before formal extraction, a pilot test was performed on 5 randomly selected studies to test form operability and revise ambiguous fields.

### 2.6 Data analysis and presentation

We used descriptive analysis without meta-analysis or statistical inference. We summarized the distribution of diagnostic criteria, measurement tool combinations, detection rate ranges, and methodological features through frequency counts, proportions, and tables. An evidence matrix presented the cross-distribution of diagnostic criteria, measurement tools, and HF types. Charts showed detection rate ranges and geographic distribution by criterion. Structured tables summarized evidence gaps. All results were presented descriptively.

## 3 Results

The systematic search retrieved 6,614 records. After deduplication and title/abstract screening, 270 papers entered full-text assessment. Twenty-three studies met the inclusion criteria. The flow diagram is in Figure 1.

**Figure 1:**
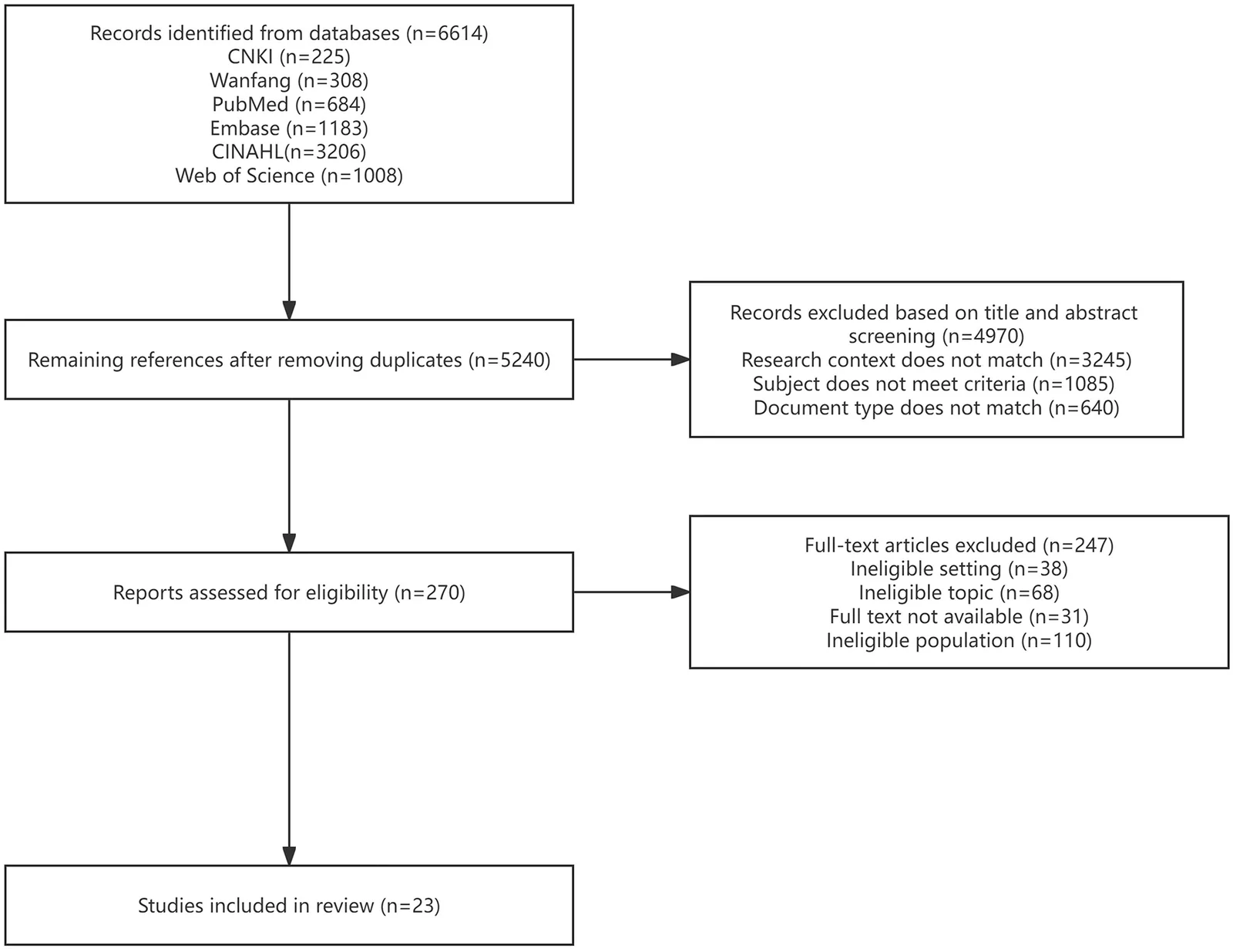
PRISMA flow diagram of literature screening and selection. The systematic search retrieved 6614 records from PubMed, Embase, CINAHL, Web of Science, CNKI, and Wanfang. After deduplication and title/abstract screening, 270 full-text articles were assessed for eligibility. Twenty-three studies met the inclusion criteria and were included in this scoping review. CNKI, China National Knowledge Infrastructure.

### 3.1 Basic characteristics of included studies

The 23 included studies were published from 2019 to 2026; 15 were published in 2023 or later (21, 26, 27, 29–31, 33–35, 37–42). Geographic distribution was mainly Asia (13 studies), including 11 from Japan (28–30, 32, 33, 36–40, 42) and 2 from China (35, 43); Europe (5 studies), including 2 from France (22, 25) and 3 from Spain (26, 27, 34); and Brazil (5 studies) (21, 23, 24, 31, 41). Study designs included 11 cross-sectional studies (23, 24, 26, 29–31, 33–35, 41, 43), 7 retrospective cohort studies (21, 22, 25, 36–39), 3 prospective cohort studies (28, 32, 42), 1 RCT (27), and 1 ambispective observational study (40). Sample sizes ranged from 38 to 1282; the median was 212.

For HF type, 7 studies reported stratified data for HFrEF and HFpEF (21, 26, 34, 39–42). One study enrolled patients with stage B pre-HF (38), and the remaining 15 studies included unclassified or mixed symptomatic HF populations (22–25, 27–33, 35–37, 43). Because stage B HF is conceptually distinct from symptomatic stages C-D HF, findings from this cohort were interpreted separately. For NYHA class, 5 studies were mainly NYHA I-II (21–23, 31, 41), 2 were mainly NYHA III-IV (32, 34), and the remaining 16 had mixed classes or did not report. For measurement timing, 9 studies assessed at discharge or after diuresis in a euvolemic state (22, 25, 28, 32, 36, 37, 39, 42, 43), 8 in the outpatient stable phase (21, 23, 24, 26, 27, 31, 38, 41), and 6 during hospitalization after HF stabilization (29, 30, 33–35, 40). No study assessed sarcopenia at peak acute decompensation (at admission, before diuresis). See Table 1.

**Table 1:** Basic characteristics of included studies.

Author/Year	Country	Design	Sample Size	HF Type	NYHA Class	Timing	Criteria	Mass Tool	Strength Tool	Function Tool	Prevalence
Bieger P, (21)	Brazil	Cohort	106	HFrEF 71%, HFpEF 9%	I–II 82%	Outpatient stable	EWGSOP2	BIA	Grip strength	Gait speed, 6MWD, chair stand	23.6%
Blanquet M, (22)	France	Prospective longitudinal	118	Not classified	I 19%, II 57%, III 22%, IV 3%	Post-diuresis/near discharge	EWGSOP1 + EWGSOP2	BIA	Grip strength	4 m gait speed	EWGSOP1: 38.1%; EWGSOP2 confirmed: 24.6%
Canteri AL, (23)	Brazil	Cross-sectional + control	222 (79 HF)	HFrEF (LVEF≤40%)	I 9%, II 62%, III 29%	Stable outpatient	FNIH	DXA	Grip strength	4 m gait speed	10.1%
da Fonseca GWP, (24)	Brazil + Germany	Cross-sectional (matched RCT subsample)	64	HFrEF (LVEF≤40%)	II/III	Stable chronic HF	EWGSOP2	DXA	Grip strength	Peak VO₂ (not for diagnosis)	23.4%
Eschalier R, (25)	France	Multicenter prospective longitudinal + validation cohort	140 (main) + 165 (validation)	Mixed (48.9% LVEF<40%)	I 21%, II 52%, III 24%, IV 3%	Post-diuresis/discharge/rehab	EWGSOP1	BIA	Grip strength	4 m gait speed	65.0% (95%CI 57.1–72.9%)
Guillén RL, (26)	Spain	Cross-sectional, single-center	121	HFrEF 35%, HFmrEF 19%, HFpEF 43%	I 43%, II 33%, III 22%, IV 3%	Stable outpatient	EWGSOP2	BIA + Ultrasound	Grip strength	SPPB	33.1% (confirmed 20.7% + severe 12.4%)
Herrera-Martínez AD, (27)	Spain	RCT	38	Not classified (LVEF<50%)	NR	Stable/ambulatory (HF hospitalization within 6mo)	Custom	BIA	Grip strength (age/gender-adjusted ≤ p25)	TUG	65.8%
Honda S, (28)	Japan	Retrospective cohort	272	Mixed (HFpEF 35%)	NR	Pre-discharge euvolemic	AWGS 2019	BIA	Grip strength	Gait speed	23.2%
Honma S, (29)	Japan	Ambispective cross-sectional	587	Mixed (large proportion preserved LVEF)	Mixed (III: sarcopenia 36% vs non 19%)	Inpatient (cardiac rehab)	AWGS 2019	DXA	Grip strength	Gait speed, FTSS, SPPB	58.8%
Kawaharata W, (30)	Japan	Single-center cross-sectional	212 (DXA–CT); 186 (sarcopenia)	HF (ESC 2016 criteria)	Mixed; III/IV 32% overall, 29% subgroup	Post-stabilization inpatient	AWGS 2019	DXA + CT	Grip strength	SPPB	35.0%
Knobloch IdS, (31)	Brazil	Cross-sectional	151	Not classified (76% HFrEF)	I–II 84%, III–IV 16%	Stable	EWGSOP2	Anthropometry	Grip strength (Jamar, non-dominant, 3 trials)	NR	36.1%
Kono Y, (32)	Japan	Prospective observational	186	Not classified (ischemic 28%, dilated 31%, hypertensive 25%)	III 92% at admission	Post-ADHF, pre-discharge	Custom	Anthropometry	Grip strength (Jamar, seated, 3 trials/hand)	10 m gait speed	41.2%
Kouzu H, (33)	Japan	Cross-sectional	384	Not classified	Mixed	Stable	AWGS 2019	DXA	NR	NR	58%
Lorido JCA, (34)	Spain	Single-center observational	224	HFpEF 71%, HFrEF 18%, HFmrEF 11%	II 21%, III 67%, IV 10%	Post-stabilization after ADHF admission	EWGSOP2	Anthropometry	Grip strength	TUG + SPPB (not for diagnosis)	11.2% (ASMI); 8.9% (calf circumference)
Lu M, (35)	China	Cross-sectional	180	HFpEF	NR	NR	AWGS 2019	BIA	Grip strength	Gait speed + chair stand	43.9%
Maeda D, (36)	Japan	Prospective cohort (FRAGILE-HF post-hoc)	1,332 (960 assessed)	Not classified (Framingham criteria)	Baseline distribution reported	Stable	AWGS 2014	BIA	Grip strength (dynamometer)	4 m gait speed	19.7%
Maeda D, (37)	Japan	Prospective multicenter registry post-hoc	1,262 (analysis)/1,332 (registered)	Mixed (LVEF median 45% [31–60])	III–IV 14.6% overall	Post-diuresis stable, pre-discharge	AWGS 2014	BIA	Grip strength (TKK 5101)	4 m gait speed	19.7% (184/936)
Matsumura K, (38)	Japan	Prospective multicenter observational	312 (331 registered, 19 excluded)	Stage B HF (ARIC criteria: structural HD/ cardiac dysfunction, asymptomatic)	NA (Stage B = asymptomatic, no NYHA)	Stable outpatient (no HF hospitalization, no diuretics)	AWGS 2019	BIA	Grip strength (GRIP-D)	6 m gait speed/chair stand/SPPB (any one)	17.7%
Nakade T, (39)	Japan	Cohort (prospective multicenter post-hoc)	1,282	HFrEF + HFpEF mixed	Mixed	Stable pre-discharge	SDOC + AWGS 2019	BIA	Grip strength	4 m gait speed/SPPB/chair stand	SDOC: 61.3%; AWGS 2019: 23.0%
Ohori K, (40)	Japan	Single-center ambispective	447	Mixed; 48% LVEF<50%, 52% LVEF≥50%	I–II ∼72%, III 28%	Mixed; 28% ADHF, assessed after symptom relief	AWGS 2019	DXA	Grip strength (Smedley dynamometer, standing, 2 trials/hand)	10 m gait speed	51%
Sangali TD, (41)	Brazil	Cross-sectional	90	89% HFrEF, 11% HFpEF	I–II 78%, III–IV 22%	Stable outpatient (diagnosed ≥3 months)	EWGSOP2	BIA	Grip strength (Jamar, dominant hand, seated 90° elbow flexion) + chair stand	6 m gait speed	24.4%
Shakuta S, (42)	Japan	Retrospective cohort	546	HFrEF 348, HFpEF 187	I–II/III–IV mixed (III/IV 18.5%)	Post-ADHF pre-discharge baseline → post-cardiac rehab (3–5mo)	AWGS 2019	Anthropometry	Grip strength (TKK 5101, seated 90°, 2 trials/hand)	10 m gait speed + SPPB + chair stand	30.9%
Zhao W, (43)	China (Nanjing)	Cross-sectional	355	Mixed (criteria covered HFrEF and HFpEF)	NR	Stable/pre-discharge	AWGS 2014	BIA	Grip strength (Xiangshan mechanical, dominant hand, seated, 2 trials)	4 m gait speed	55.8%

HFrEF, heart failure with reduced ejection fraction; HFmrEF, heart failure with mildly reduced ejection fraction; HFpEF, heart failure with preserved ejection fraction; NYHA, New York Heart Association; NR, not reported; BIA, bioelectrical impedance analysis; DXA, dual-energy x-ray absorptiometry; CT, computed tomography; MRI, magnetic resonance imaging; 6MWD, 6 min walk distance; SPPB, Short Physical Performance Battery; FTSS, five-times sit-to-stand; TUG, Timed Up and Go. Overall prevalence calculated using each study's preferred definition; when both possible and confirmed sarcopenia reported, confirmed value used; when both sarcopenia and severe sarcopenia reported, overall sarcopenia value used. Mixed HF type or unstratified HFrEF/HFpEF reported as “not classified” or “mixed”; subgroup sample sizes in parentheses.

### 3.2 Distribution of diagnostic criteria and measurement tools

The included literature covered 8 diagnostic criteria or criterion combinations: AWGS 2019, EWGSOP2, AWGS 2014, EWGSOP1, FNIH, SDOC, and 2 custom criteria. GLIS and the International Working Group on Sarcopenia (IWGS) criteria were not used in the included literature. AWGS 2019 was most common (9 studies) (28–30, 33, 35, 37–39, 42), followed by EWGSOP2 (7 studies) (21, 22, 24, 26, 31, 34, 41), AWGS 2014 (3 studies) (36, 37, 43), EWGSOP1 (2 studies) (22, 25), SDOC (1 paper) (39), FNIH (1 paper) (23), and custom criteria (2 studies) (27, 32). Three studies performed head-to-head comparisons: one applied both EWGSOP1 and EWGSOP2 in the same cohort, showing EWGSOP1 detection rate (38.1%) was higher than EWGSOP2 confirmed rate (24.6%) (22); one compared SDOC and AWGS 2019, finding SDOC detection rate (61.3%) was higher than AWGS 2019 (23.0%) (39); one compared the SARC-F, Mini Sarcopenia Risk Assessment-7 (MRSA-7), and Mini Sarcopenia Risk Assessment-5 (MRSA-5) screening tools against AWGS 2014 for diagnostic accuracy (43). See Table 2A.

**Table 2A:** Diagnostic dimensions and cutoff settings.

Author/Year	Diagnostic Dimensions	Muscle Mass Cutoff (Male)	Muscle Mass Cutoff (Female)	Muscle Strength Cutoff (Male)	Muscle Strength Cutoff (Female)	Function Cutoff	Head-to-Head
Bieger P, (21)	2D (strength + mass) confirmed; 3D (+function) severe	ASMM <20 kg	ASMM <15 kg	Grip <27 kg	Grip <16 kg	Gait speed <0.8 m/s; 6MWD <300 m; chair stand >15 s	No
Blanquet M, (22)	EWGSOP1: 2D (mass + strength/function); EWGSOP2: 2D (probable: strength only; confirmed: strength + mass)	SMI <8.6 kg/m^2^ (French reference, 2SD below young adult mean)	SMI <6.2 kg/m^2^ (French reference)	EWGSOP1: <30 kg; EWGSOP2: <27 kg; Screening: <27 kg	EWGSOP1: <20 kg; EWGSOP2: <16 kg; Screening: <18 kg	4 m gait speed <0.8 m/s	Yes (EWGSOP1 vs EWGSOP2)
Canteri AL, (23)	3D (mass + strength + function)	ALM/BMI <0.789	ALM/BMI <0.512	Grip <26 kg or HGS/BMI <1	Grip <16 kg or HGS/BMI <0.56	Gait speed ≤0.8 m/s	No
da Fonseca GWP, (24)	2D (mass + strength); function not used for diagnosis	ALM/height^2^ < 7.0 kg/m^2^	NA (males only)	EWGSOP2 default (not reported)	—	Not used for diagnosis	No
Eschalier R, (25)	2D (mass + function/strength, algorithm: gait speed→grip→mass)	MMI <10.75 kg/m^2^	MMI <6.75 kg/m^2^	Grip <30 kg	Grip <20 kg	Gait speed ≤0.8 m/s	No
Guillén RL, (26)	3D (strength + mass/quality + function, full EWGSOP2)	ASM <20 kg or ASMI <7.0 kg/m^2^	ASM <15 kg or ASMI <5.5 kg/m^2^	Grip <27 kg or 30-s chair stand >15 s	Grip <16 kg or 30-s chair stand >15 s	SPPB ≤8 (for severe sarcopenia)	No
Herrera-Martínez AD, (27)	1D (strength only)	NA (not used)	NA	Age/gender-adjusted ≤p25 (kg not reported)	Age/gender-adjusted ≤p25 (kg not reported)	—	No
Honda S, (28)	3D (mass + strength + function)	ASMI <7.0 kg/m^2^	ASMI <5.7 kg/m^2^	Grip <28 kg	Grip <18 kg	Gait speed <1.0 m/s	No
Honma S, (29)	3D (mass + strength + function)	ASMI <7.00 kg/m^2^	ASMI <5.40 kg/m^2^	Grip <28 kg	Grip <18 kg	Gait speed <1.0 m/s; FTSS ≥12 s; SPPB ≤9	No
Kawaharata W, (30)	3D (mass + strength + function)	ASMI <7.0 kg/m^2^ (AWGS 2019)	ASMI <5.4 kg/m^2^	Grip <28 kg	Grip <18 kg	SPPB ≤9	No
Knobloch IdS, (31)	2D (strength + mass)	CC ≤34 cm; AMC adequacy <90%	CC ≤33 cm; AMC adequacy <90%	≤27 kg	≤16 kg	NR	No
Kono Y, (32)	3D indices (BMI + grip + gait speed), ≥2 criteria	BMI <18.5 kg/m^2^	BMI <18.5 kg/m^2^	<26 kg	<18 kg	<0.8 m/s	No
Kouzu H, (33)	1D (mass only)	ASMI <7.0 kg/m^2^	ASMI <5.4 kg/m^2^	NR	NR	NR	No
Lorido JCA, (34)	2D (mass + strength)	ASMI: NR; Calf circumference <31 cm	ASMI: NR; Calf circumference <31 cm	Grip <27 kg	Grip <16 kg	Not used for diagnosis	No
Lu M, (35)	3D (mass + strength + function)	ASMI <7.0 kg/m^2^	ASMI <5.7 kg/m^2^	<28 kg	<18 kg	Gait speed <1.0 m/s or chair stand ≥12 s	No
Maeda D, (36)	3D (mass + strength + function)	ASMI ≤7.0 kg/m^2^	ASMI ≤5.7 kg/m^2^	<26 kg	<18 kg	4 m gait speed ≤0.8 m/s	No
Maeda D, (37)	3D (mass + strength + function)	ASM/ht^2^ ≤ 7.0 kg/m^2^	ASM/ht^2^ ≤ 5.7 kg/m^2^	Grip <26 kg	Grip <18 kg	4 m gait speed <0.8 m/s	No
Matsumura K, (38)	3D (mass + strength + function)	ASM/ht^2^ < 7.0 kg/m^2^	ASM/ht^2^ < 5.7 kg/m^2^	Grip <28 kg	Grip <18 kg	6 m gait speed <1.0 m/s or chair stand >12.0 s or SPPB ≤9	No
Nakade T, (39)	SDOC: 2D (strength + function); AWGS 2019: 3D (mass + strength + function)	ASMI <7.0 kg/m^2^	ASMI <5.7 kg/m^2^	Grip <28 kg	Grip <18 kg	Gait speed <1.0 m/s/SPPB/chair stand	Yes (SDOC vs AWGS 2019)
Ohori K, (40)	3D (mass + strength + function)	ASM/ht^2^ < 7.0 kg/m^2^	ASM/ht^2^ < 5.4 kg/m^2^	Grip <28 kg	Grip <18 kg	10 m gait speed <1.0 m/s	No
Sangali TD, (41)	3D (mass + strength + function)	ASM <20 kg or ASM/ht^2^ < 7.0 kg/m^2^	ASM <15 kg or ASM/ht^2^ < 5.5 kg/m²	Grip <27 kgf; chair stand >15 s	Grip <16 kgf; chair stand >15 s	Gait speed ≤0.8 m/s	No
Shakuta S, (42)	3D (mass + strength + function)	ASMI <7.0 kg/m^2^	ASMI <5.4 kg/m^2^	Grip <28 kg	Grip <18 kg	Gait speed <1.0 m/s; SPPB ≤9; chair stand ≥12 s	No
Zhao W, (43)	3D (mass + strength + function)	NR (AWGS 2014 default: ASM/ht^2^<7.0)	NR (AWGS 2014 default: ASM/ht^2^<5.4)	NR (AWGS 2014 default: <26 kg)	NR (AWGS 2014 default: <18 kg)	NR (AWGS 2014 default: <0.8 m/s)	Yes (SARC-F/MRSA-7/MRSA-5 vs AWGS 2014)

ASMM, appendicular skeletal muscle mass; ALM, appendicular lean mass; SMI, skeletal muscle index; MMI, muscle mass index; AMC, arm muscle circumference; CC, calf circumference; HGS, handgrip strength; BIA, bioelectrical impedance analysis; DXA, dual-energy x-ray absorptiometry; SPPB, Short Physical Performance Battery; TUG, Timed Up and Go; FTSS, five-times sit-to-stand; 6MWD, 6 min walk distance; NR, not reported; NA, not applicable. Cutoffs not explicitly reported in the main text and using standard default recommended values are labeled with the standard name; cutoffs customized or based on specific reference populations are labeled as “custom” or “study-specific.” Head-to-head comparison refers to studies applying two or more sarcopenia diagnostic criteria in the same cohort and reporting respective detection rates or diagnostic performance.

### 3.3 Detection rate differences

All 23 studies reported overall prevalence. Detection rates varied within the same criterion. For AWGS 2019, the range was 17.7% (outpatient stage B HF) (38) to 58.8% (inpatients) (29). For EWGSOP2, the range was 8.9% (based on calf circumference, stable hospitalized phase) (34) to 36.1% (outpatient stable phase) (31); other studies reported 23.4% (stable chronic HF) (24), 23.6% (outpatient stable phase) (21), 24.4% (outpatient stable phase) (41), and 33.1% (outpatient stable phase) (26). EWGSOP1 had relatively lenient thresholds, with detection rates at high levels (38.1% to 65.0%) (22, 25). Head-to-head comparisons showed SDOC detection rate (61.3%) was higher than AWGS 2019 (23.0%) (39), and EWGSOP1 (38.1%) was higher than EWGSOP2 (24.6%) (22).

Stratified data were limited. Eight studies reported sex-stratified prevalence (22, 23, 25, 29, 36, 37, 40, 42). Seven reported HF type (HFrEF/HFpEF) stratified data (21, 26, 34, 39–42). Four reported NYHA class stratified data (21, 22, 25, 29). Data directly comparing acute and stable phases in the same patients were scarce. One study reported a 65.0% detection rate after acute decompensation at discharge (25), whereas the stage B pre-HF outpatient cohort reported a detection rate of 17.7% (38). These figures are not directly comparable because patient severity, diagnostic criteria, measurement tools, and clinical setting differed substantially. They may, in part, reflect differences in measurement timing, but no timing-specific effect can be inferred from these cross-study data.

### 3.4 Barriers from heart failure-specific factors

Discussion of HF-specific barriers was limited in existing studies. Five studies addressed edema effects on sarcopenia assessment; 4 discussed this explicitly (21, 22, 25, 43), and 1 addressed it indirectly (34). None reported specific BIA edema correction methods or formulas. Some studies noted BIA should be performed after stopping intravenous diuretics in a euvolemic state (22), or excluded patients who had not reached euvolemia (25), or combined BIA with ultrasound to avoid edema interference (34). Most studies did not mention edema.

For cardiac function limitation, 3 studies discussed limits on completing function tests (22, 23, 25). One study reported 5 patients were excluded because they could not complete 4-meter gait speed and grip strength tests; the completion rate was 96.4% (25). Another excluded patients unable to complete 4-meter gait speed; NYHA class IV accounted for 2.5% in this cohort (22). Most studies did not report the proportion of NYHA class IV patients, function test completion rates, or alternative assessment plans.

For acute phase timing, no paper diagnosed sarcopenia at peak acute decompensation (at admission, before diuresis). Six studies assessed during hospitalization after HF stabilization (29, 30, 33–35, 40). Nine studies assessed at discharge or after diuresis in a stable state (22, 25, 28, 32, 36, 37, 39, 42, 43). Eight studies assessed in the outpatient stable phase (21, 23, 24, 26, 27, 31, 38, 41). Weight fluctuation and cut-off applicability timing were not discussed. No study reported dry weight vs. admission weight comparison data.

Test safety and completion rate reporting was limited. Four studies reported function test completion rates (25, 30, 31, 37): 96.4% (25), 87.7% (30), 95.4% (31), and 74.2% (37). No paper reported adverse events during sarcopenia assessment, such as arrhythmia, hypotension, or falls. See Table 2B.

**Table 2B:** HF-specific considerations, prognostic association and predictive performance, and evidence gaps.

Author/Year	Timing	HF-Specific Considerations	Prognostic Association and Predictive Performance	Evidence Gaps
Bieger P, (21)	Outpatient stable	Edema: excluded edematous patients; no BIA correction method. Function: NYHA III–IV 18%, but outpatient tests feasible. Completion: NR	3-yr follow-up; outcomes: all-cause/HF hospitalization, all-cause/HF death; PA < 5.45° predicted HF hospitalization HR = 2.28 (1.03–5.02)	No ADHF data; limited HFpEF data; PA cutoff needs external validation; no head-to-head comparison of AWGS/GLIS/FNIH
Blanquet M, (22)	Post-diuresis/near discharge/rehab start	Edema: BIA at normal hydration, after IV diuretics stopped. Function: excluded unable to complete 4 m gait speed; NYHA IV 2.5%. Completion: NR	Not reported	First study validating HGS cutoff in HF; no acute-phase data; no HF subtype subgroups; insufficient female sample
Canteri AL, (23)	Stable outpatient	Edema: not discussed. Function: indirectly discussed—low gait speed associated with RV dysfunction (*P* = 0.016) and lower LVEF (*P* = 0.037). Completion: NR	Not reported	No acute-phase data; no HFpEF data; no discussion of edema interference with DXA; no BIA/phase angle used
da Fonseca GWP, (24)	Stable chronic HF	Edema: not discussed. Function: not discussed. Completion: NR	Peak VO₂ (functional capacity); ALM OR = 1.179 (1.011–1.374), *P* = 0.035	Males only; small sample; long-term follow-up not analyzed; exercise modality differences
Eschalier R, (25)	Post-diuresis stable/pre-discharge/rehab	Edema: only clinically euvolemic patients included; measured after HF signs resolved/IV diuretics stopped/rehab start. Function: 5 excluded due to inability to complete 4 m gait speed and grip (bedridden or unable to stand). Completion: 96.4% (135/140)	Up to 48 mo (median 24); outcomes: all-cause death, CV death, non-CV death, CV/HF/non-CV hospitalization, composite; non-CV hospitalization HR = 1.93 (1.14–3.24), *P* = 0.014	First ADHF sarcopenia prevalence study; EWGSOP1 criteria; no phase angle data; no ethnicity-specific cutoffs
Guillén RL, (26)	Stable outpatient	Edema: indirectly discussed—classical parameters may be masked by edema, fluid overload, and inflammation; used BIA + ultrasound. Function: not discussed. Completion: NR	11 deaths (9.1%) during data collection; descriptive comparison only	Outpatient HF sarcopenia prevalence; EWGSOP2; BIA + ultrasound combined; no acute-phase data; no sex/HF subtype stratified prevalence
Herrera-Martínez AD, (27)	Baseline/stable outpatient	Edema: not discussed. Function: not discussed. Completion: NR	24-wk follow-up; outcomes: all-cause death, new HF hospitalization, LVEF, NT-proBNP, QoL; nutrition support OR = 0.24 (0.02–2.62); sarcopenia prognostic effect not reported	Custom 1D grip-strength criterion, not formal sarcopenia diagnosis; no mass/function cutoffs; relative percentile cutoffs rather than absolute values
Honda S, (28)	Pre-discharge euvolemic	Edema: not discussed. Function: not discussed. Completion: NR	1-yr follow-up; outcome: cardiac death or HF rehospitalization (composite); univariate HR = 2.191 (1.229–3.905); multivariate HR = 2.384 (1.305–4.354), *P* = 0.005	Single-center retrospective; AWGS 2019 cutoff not validated in HF/edema; no discussion of BIA interference in acute HF; no test completion/failure data; no HFrEF/HFpEF prevalence differentiation
Honma S, (29)	Inpatient (pre-discharge assessment)	Edema: not discussed. Function: not discussed. Completion: NR	Not reported	AWGS 2019 not validated in hospitalized HF; no NYHA IV/test completion data; no discussion of edema effect on DXA
Kawaharata W, (30)	Post-stabilization inpatient	Edema: not discussed. Function: not discussed. Completion: 87.7% (186/212 completed grip + SPPB)	Not reported	No head-to-head comparison; no HF subtype stratified prevalence; no discussion of edema/diuretic effect on body composition; CT opportunistic scanning lacks standardization
Knobloch IdS, (31)	Stable outpatient	Edema: discussed. Function: not discussed. Completion: 95.4% (144/151)	Not reported	No acute-phase data; no HFpEF stratification; no sex/age-specific prevalence reported
Kono Y, (32)	Post-ADHF, pre-discharge	Edema: not discussed. Function: not discussed. Completion: NR	Not reported	Standard lacks year/abbreviation; no edema discussion; BMI as mass proxy not validated; no test completion data
Kouzu H, (33)	Post-stabilization inpatient	Edema: not discussed. Function: not discussed. Completion: NR	Not reported	1D mass-only definition, not full sarcopenia diagnosis; no strength/function data; no HF subtype or NYHA stratification; no ADHF data
Lorido JCA, (34)	Post-stabilization after ADHF admission	Edema: discussed. Function: not discussed. Completion: NR	30-day (early readmission) + in-hospital; outcomes: 30-day readmission, in-hospital death; 30-day readmission OR 4.9 (1.2–8.0, *P* = 0.03)	ASMI cutoff not explicitly reported; no direct mass measurement (BIA/DXA/CT/MRI); single-center small sarcopenia subgroup (*n* = 25); no HF subtype/sex/age stratification; no test completion/failure data
Lu M, (35)	NR	Edema: not discussed. Function: not discussed. Completion: NR	Not reported	Single-center small sample; no sex/age/NYHA stratified prevalence; no discussion of HF-specific issues (edema, weight fluctuation, acute-phase timing)
Maeda D, (36)	Pre-discharge hemodynamically compensated	Edema: not discussed. Function: not discussed. Completion: NR	1-yr follow-up; outcome: all-cause death; male HR 1.93 (1.13–3.31); female HR 3.18 (1.59–6.32)	AWGS 2014 old version cutoffs; no HF subtype prevalence; no acute-phase/edema correction/test completion data; male grip cutoff <26 kg differs from 2019 version
Maeda D, (37)	Post-diuresis stable/pre-discharge	Edema: discussed. Function: not discussed. Completion: 74.2% (936/1,262 assessed, failure reasons not reported)	1-yr follow-up; outcome: all-cause death; High vs Low Ishii HR = 1.54 (1.06–2.25); per 10-point increase HR = 1.09 (1.03–1.16)	ADHF data; stable inpatient HF measurement but no NYHA IV or acute exacerbation direct diagnosis; no edema correction; low female AUC reliability; no HF subtype data
Matsumura K, (38)	Stable outpatient	Edema: not discussed. Function: not discussed. Completion: NR	2-yr follow-up; outcome: composite (all-cause death + MI + HF hospitalization); sarcopenia vs non HR = 5.78 (2.16–15.43)	Stage B HF-specific data; first Stage B HF sarcopenia study but small sample, few events, HR may be overestimated; no HFpEF/HFrEF subtype; no sex-stratified prevalence; no edema correction; no test completion data
Nakade T, (39)	Pre-discharge clinically stable	Edema: not discussed. Function: not discussed. Completion: NR	2-yr follow-up; outcome: all-cause death; possible sarcopenia HR = 1.22 (0.66–2.28); sarcopenia HR = 1.89 (1.08–3.34)	No ADHF data; no edema correction method; no test completion/adverse event data; Japanese elderly only, lacking external validation
Ohori K, (40)	Inpatient, post-symptom relief	Edema: not discussed. Function: not discussed. Completion: NR	Median 763 days; outcome: all-cause death; sarcopenia + weight loss vs double-negative HR≈4–5 (*P* = 0.007); C-index 0.741; cNRI 0.537; IDI 0.019	No HF subtype prevalence; no discussion of edema effect on body composition; no test completion/failure data
Sangali TD, (41)	Stable outpatient (diagnosed ≥3 months)	Edema: not discussed. Function: not discussed. Completion: NR	Not reported	EWGSOP2 not validated in HF; BIA not corrected for edema; no HF subtype stratified prevalence; no test completion data
Shakuta S, (42)	Pre-discharge (baseline) + post-cardiac rehab (3–5 mo)	Edema: not discussed. Function: not discussed. Completion: NR	Median 3.1 yr (IQR 1.3–5.0); outcomes: all-cause death, composite (all-cause death + first HF emergency readmission), total HF readmissions; unimproved vs robust: all-cause death HR = 2.603 (1.375–4.930); composite HR = 2.099 (1.349–3.264); HF readmission IRR = 1.517 (1.026–2.242)	Anthropometric prediction equation instead of BIA/DXA; no ADHF (pre-diuresis) data; no NYHA IV-specific data; no test completion/failure data
Zhao W, (43)	Stable/pre-discharge	Edema: discussed. Function: discussed. Completion: NR	Not reported	AWGS cutoff directly applied to CHF inpatients without HF-specific validation; no ADHF data; no HF subtype stratification; HD patients 52.7%, no separate subgroup prevalence reported

HR, hazard ratio; OR, odds ratio; IRR, incidence rate ratio; NR, not reported; NA, not applicable; BIA, bioelectrical impedance analysis; DXA, dual-energy x-ray absorptiometry; CT, computed tomography; MRI, magnetic resonance imaging; SPPB, Short Physical Performance Battery; TUG, Timed Up and Go; FTSS, five-times sit-to-stand; 6MWD, 6 min walk distance; CV, cardiovascular; HF, heart failure; QoL, quality of life; LVEF, left ventricular ejection fraction; NT-proBNP, N-terminal pro-B-type natriuretic peptide; AUC, area under the curve; cNRI, continuous net reclassification improvement; IDI, integrated discrimination improvement; PA, phase angle; ADHF, acute decompensated heart failure; ICFER, heart failure with reduced ejection fraction (Portuguese); ICFEP, heart failure with preserved ejection fraction (Portuguese). In the HF-specific considerations column, “edema” indicates whether the study discussed or addressed edema interference with sarcopenia assessment; “function” indicates whether NYHA class or cardiac function impact on test completion was discussed; “completion” indicates the actual completion rate of sarcopenia dimension tests. In the prognostic association and predictive performance column, studies not reporting prognosis are labeled “Not reported.” In the measurement timing column, “outpatient stable” refers to outpatient follow-up or chronic stable patients; “inpatient stable” refers to post-acute decompensation hemodynamically stable but not yet discharge-ready; “pre-discharge/post-diuresis” refers to euvolemic assessment after IV diuretics stopped or near discharge. Evidence gaps are key limitations identified by study authors or extracted by this review based on methodological reporting, used for evidence mapping in the Discussion.

### 3.5 Prognostic association and predictive performance

Eleven studies reported prognostic associations (21, 25, 27, 28, 34, 36–40, 42), but reporting of model discrimination, calibration, and reclassification was limited. Cox proportional-hazards models showed associations between sarcopenia and death or rehospitalization; these adjusted associations should not be interpreted as predictive-performance metrics. Only 1 study reported advanced predictive metrics, including a concordance index (C-index) of 0.741, continuous net reclassification improvement (cNRI) of 0.537, and integrated discrimination improvement (IDI) of 0.019 (40). Another study compared the area under the receiver operating characteristic curve (AUROC) between SDOC and AWGS 2019, with no statistically significant difference (39). Most studies did not report calibration or conduct formal external validation of diagnostic and prognostic performance. Although some studies established independent multicenter validation cohorts (25) or used multicenter registry databases (37–39), external validation of the diagnostic criteria themselves remained insufficient.

## 4 Discussion

The above results present the current application of sarcopenia diagnostic criteria in HF patients, the distribution of measurement tools, and preliminary data on prognostic association and predictive performance. However, existing evidence shows clear gaps in HF-specific validation of diagnostic criteria, hydration state correction for measurement tools, acute phase assessment timing, and alternative approaches for severely ill patients. The following sections discuss the applicability of diagnostic criteria, measurement tool selection, implementation barriers from HF-specific factors, and the current state of prognostic association and predictive-performance assessment, to inform clinical practice and future research.

### 4.1 Resource identification initiative

AWGS 2019 and EWGSOP2 are the two most common diagnostic frameworks in HF populations (21, 22, 24, 26, 28–31, 33–35, 37–39, 41, 42). Table 3 summarizes how the main diagnostic frameworks combine muscle strength, muscle mass, and physical performance (10, 11, 13). These frameworks differ in the dimensions required, measurement tools, reference populations, and cut-off values; consequently, variation in reported prevalence under the same diagnostic label may reflect both true population differences and methodological differences in how sarcopenia is operationalized. Detection rates within the same criterion vary widely: AWGS 2019 spans from 17.7% to 58.8% (29, 38), and EWGSOP2 shows a difference of nearly 12 percentage points (31, 41). This heterogeneity stems not only from differences in patient age and HF severity but also from the direct application of criteria developed in general older adults without adequate HF-specific validation. EWGSOP1 used relatively lenient thresholds and a different case-finding approach, yielding systematically higher detection rates (22, 25). SDOC places greater emphasis on muscle function than on muscle mass and produces higher positive rates (39), but whether its long-term prognostic performance equals that of muscle-mass-dependent criteria remains unresolved. Only 3 papers performed criterion-to-criterion head-to-head comparisons (22, 39, 43), and all used single-center designs. Current evidence is therefore insufficient to establish the superiority of any one diagnostic criterion in HF populations.

**Table 3:** Summary of main diagnostic frameworks applied in heart failure studies.

Framework	Muscle strength	Muscle mass	Physical performance	Operational definition
EWGSOP2 (11)	Central and primary indicator	Required for confirmed and severe sarcopenia	Required for severe sarcopenia	Probable sarcopenia: low strength; confirmed sarcopenia: low strength plus low muscle mass; severe sarcopenia: low strength, low muscle mass, and low physical performance
AWGS 2019 (13)	One of the two function-related dimensions	Required	Alternative function-related dimension to low strength	Sarcopenia: low muscle mass plus low strength or low physical performance; severe sarcopenia: low muscle mass, low strength, and low physical performance
EWGSOP1 (10)	Alternative to low physical performance after low muscle mass is identified	Required	Alternative to low strength after low muscle mass is identified	Low muscle mass plus low strength or low physical performance; compared with EWGSOP2, EWGSOP1 uses a different case-finding algorithm and relatively broader thresholds

This table summarizes how diagnostic dimensions are combined and does not reproduce every sex-, device-, or population-specific cut-off value. Exact thresholds vary according to the measurement tool and reference population used.

### 4.2 Heart failure-specific limits of measurement tools

BIA is the main tool for muscle mass assessment (13 papers) (21, 22, 25, 26, 28, 31, 33, 35–39, 43). Its accuracy depends heavily on hydration state. HF patients often have non-euvolemic status because of volume load fluctuation and diuretic use. In existing studies, only a few explicitly excluded patients with obvious edema or required measurement after stopping intravenous diuretics (21, 22, 25). No study used a validated edema correction formula. DEXA is regarded as the reference standard, but equipment availability, cost, and transport requirements limit its routine use in acute HF wards (23, 24, 29, 30, 40). Computed tomography (CT) opportunistic measurement shows high diagnostic accuracy, with area under the curve (AUC) up to 0.96 (30), but lacks standardized protocols for HF populations, and radiation exposure limits its use as a first-line screening tool. Grip strength dominates muscle strength assessment (22 papers) (21–32, 34–43). This choice is logical: the test is simple and low-cost. Yet grip strength can be affected by positional limits in NYHA class IV patients, upper limb edema, and joint disease. For function assessment, gait speed is most common (16 papers) (21–23, 25, 28, 29, 32, 35–43), but 4-meter or 6-meter tests are often hard to complete during acute decompensation. SPPB and FTSS provide multidimensional information, yet their completion rates and safety data in HF patients are under-reported (25, 30, 31, 37).

These between-study differences in devices, measurement conditions, and functional tests further complicate cross-study comparison because the same diagnostic label may be based on different estimates of muscle mass or different performance measures.

### 4.3 Barriers in acute decompensation and severe patients

Acute decompensation may be an important window for sarcopenia screening. Yet this review found no study that assessed sarcopenia before diuresis or at admission. Measurement timing clustered in three periods: during hospitalization after stabilization (6 papers) (29, 30, 33–35, 40); at discharge or after diuresis in a euvolemic state (9 papers) (22, 25, 28, 32, 36, 37, 39, 42, 43); and in the outpatient stable phase (8 papers) (21, 23, 24, 26, 27, 31, 38, 41). No study reached peak acute decompensation. One study reported a high detection rate of 65.0% at discharge (25), while another reported 17.7% in outpatient stable stage B HF (38). The 65.0% discharge cohort and the 17.7% stage B pre-HF outpatient cohort differed in HF stage and severity, diagnostic framework, measurement tools, and clinical setting. These figures are therefore descriptive and cannot isolate the effect of measurement timing. Nevertheless, the complete absence of pre-diuresis assessment means that acute-phase feasibility, prevalence, and safety remain unknown.

NYHA class IV patients are more challenging. This group is often excluded because they cannot stand or complete standard gait speed tests. Some studies reported only a few cases excluded because of bed rest or inability to stand (25); in another study, NYHA class IV accounted for less than 3% (22). Alternative assessment options, whether bedside muscle strength tests, ultrasound muscle measurement, or phase angle assessment, lack evidence-based support (21, 30).

### 4.4 Data gaps in test safety and completion rates

Test safety data are almost absent. Four papers reported the proportion of patients who completed function tests (25, 30, 31, 37), ranging from 74.2% to 96.4%. No paper recorded adverse events during assessment, such as arrhythmia, hypotension, or falls. These are real risks in acute HF patients. This data gap limits the spread of screening tools in acute HF populations.

### 4.5 Gaps in prognostic association, predictive performance, and external validation

Eleven studies reported associations between sarcopenia and death or rehospitalization (21, 25, 27, 28, 34, 36–40, 42). Hazard ratios derived from Cox proportional-hazards models indicate associations, often after adjustment for covariates, but do not by themselves establish model discrimination, calibration, or incremental predictive value. Only 1 study reported a C-index of 0.741, cNRI of 0.537, and IDI of 0.019 (40), and another compared AUROC between SDOC and AWGS 2019, with no statistically significant difference (39). Calibration was rarely reported. Although some studies established independent multicenter validation cohorts (25) or used multicenter registry databases (37–39), external validation of both diagnostic performance and prognostic transportability remained insufficient. The generalizability of current cut-off values in HF populations therefore remains uncertain.

### 4.6 Evidence gaps and future research directions

This review identified several gaps. No validated HF-specific sarcopenia diagnostic cut-off values were identified. BIA edema correction methods, alternative assessment options for NYHA class IV patients, acute decompensation diagnostic strategies, and test safety data are all absent. In addition, implementation science metrics such as time required for assessment, test tolerance across NYHA classes, and inter-rater agreement have rarely been measured (22, 25, 31, 37). The diagnostic value and safety of alternative indicators such as phase angle, CT muscle mass, and bedside ultrasound in the acute phase need further exploration (21, 30). Future research should prioritize multicenter prospective cohorts, use clinical outcomes as reference, perform HF-specific external validation of existing criteria, establish HF-specific diagnostic cut-off values, report test completion rates and safety data systematically, and evaluate both independent prognostic associations and incremental predictive performance of different criteria in HF populations.

## 5 Limitations

The limitations of this review stem from its scoping review design and the methodological heterogeneity of included literature. Scoping reviews allow broad evidence mapping but do not include formal quality appraisal. Most included studies were observational, with wide sample size ranges, and incomplete reporting of HF type and NYHA stratification, which increases the risk of selection bias. Despite these limits, this review is the first to systematically describe the application of sarcopenia diagnostic criteria in HF patients and to map evidence gaps, providing direction for future research.

Finally, the inclusion of one stage B pre-HF cohort, together with incomplete reporting of HF phenotype and NYHA class, may increase clinical heterogeneity. Prevalence estimates were therefore interpreted descriptively rather than as evidence of timing- or criterion-specific effects.

## 6 Conclusion

Early identification of sarcopenia and targeted intervention may improve functional status and lower the risk of adverse events in HF patients. Current evidence shows that AWGS 2019 and EWGSOP2 are the most common diagnostic frameworks in clinical use, yet both were transplanted directly from general older adults. Their applicability in acute HF or during volume fluctuation has not been fully tested. Pending stronger HF-specific evidence, a pragmatic clinical approach is to consider sarcopenia assessment when patients are hemodynamically stable and approaching euvolemia, using bedside-feasible tools appropriate to the patient's condition. For bedridden patients who cannot complete standard gait-speed tests or body-composition analysis, grip strength may be used as a supplementary screening measure, but it should not be the sole diagnostic basis. Findings from stage B pre-HF populations should not be generalized directly to patients with symptomatic HF. Future research should focus on establishing HF-specific sarcopenia diagnostic cut-off values, exploring assessment timing and feasible options during acute decompensation, systematically recording function-test completion rates and adverse events, and validating the diagnostic value and safety of alternative indicators such as phase angle, bedside ultrasound, and CT muscle measurement. Such studies should also externally validate diagnostic performance and evaluate both independent prognostic associations and incremental predictive value. This evidence would help determine whether sarcopenia screening can be integrated safely and effectively into routine HF care.
