# Supplementary material for: Application and evidence gaps of sarcopenia diagnostic criteria in patients with heart failure: a scoping review
Source: Front Med (Lausanne). 2026 Sep 29;13:1957639. doi: 10.3389/fmed.2026.1957639 (PMC13659442; doi:10.3389/fmed.2026.1957639)
Supplement: Supplementary file 1 [file Table1.docx]

| **Pubmed** |  |
| --- | --- |
| #1 | "Heart Failure"[Mesh] OR "Heart Failure, Systolic"[Mesh] OR "Heart Failure, Diastolic"[Mesh] OR "heart failure"[tiab] OR HFrEF[tiab] OR HFpEF[tiab] OR HFmrEF[tiab] OR "cardiac failure"[tiab] |
| #2 | "Sarcopenia"[Mesh] OR "sarcopenia"[tiab] OR "muscle wasting"[tiab] OR "skeletal muscle atrophy"[tiab] OR "low muscle mass"[tiab] OR "reduced muscle mass"[tiab] |
| #3 | "Diagnosis"[Mesh] OR "Diagnostic Criteria"[Mesh] OR "Mass Screening"[Mesh] OR "Prevalence"[Mesh] OR "diagnostic criteria"[tiab] OR "screening"[tiab] OR "detection rate"[tiab] OR "cutoff value"[tiab] OR "cut-off"[tiab] OR "diagnostic tool"[tiab] |
| #4 | #1 AND #2 AND #3 |
| **Web of Science** |  |
| #1 | TS=("heart failure" OR "cardiac failure" OR HFrEF OR HFpEF OR HFmrEF OR "heart decompensation") |
| #2 | TS=(sarcopenia OR "muscle wasting" OR "skeletal muscle atrophy" OR "low muscle mass" OR "reduced muscle mass" OR "muscle mass loss") |
| #3 | TS=(diagnosis OR "diagnostic criteria" OR screening OR prevalence OR "detection rate" OR cutoff OR "cut-off" OR "diagnostic standard" OR "diagnostic tool") |
| #4 | #1 AND #2 AND #3 |
| **Embase** |  |
| #1 | ‘heart failure’/exp OR 'heart failure with reduced ejection fraction'/exp OR 'heart failure with preserved ejection fraction'/exp OR 'heart failure':ti,ab OR HFrEF:ti,ab OR HFpEF:ti,ab OR HFmrEF:ti,ab OR 'cardiac failure':ti,ab |
| #2 | 'sarcopenia'/exp OR 'sarcopenia':ti,ab OR 'muscle wasting':ti,ab OR 'skeletal muscle atrophy':ti,ab OR 'low muscle mass':ti,ab OR 'reduced muscle mass':ti,ab |
| #3 | 'diagnosis'/exp OR 'diagnostic criterion'/exp OR 'prevalence'/exp OR 'diagnostic criteria':ti,ab OR 'screening':ti,ab OR 'detection rate':ti,ab OR 'cutoff':ti,ab OR 'cut-off':ti,ab OR 'diagnostic tool':ti,ab |
| #4 | #1 AND #2 AND #3 |
| CINAHL |  |
| S1 | (MH "Heart Failure+") OR (MH "Heart Failure, Systolic+") OR (MH "Heart Failure, Diastolic+") OR TI(heart failure OR cardiac failure OR HFrEF OR HFpEF OR HFmrEF) OR AB(heart failure OR cardiac failure OR HFrEF OR HFpEF) |
| S2 | (MH "Sarcopenia+") OR TI(sarcopenia OR "muscle wasting" OR "skeletal muscle atrophy" OR "low muscle mass") OR AB(sarcopenia OR "muscle wasting" OR "skeletal muscle atrophy") |
| S3 | (MH "Diagnosis+") OR (MH "Diagnostic Criteria+") OR (MH "Mass Screening+") OR (MH "Prevalence+") OR TI(diagnosis OR "diagnostic criteria" OR screening OR prevalence OR "detection rate" OR cutoff) OR AB(diagnosis OR "diagnostic criteria" OR screening OR prevalence) |
| S4 | S1 AND S2 AND S3 |
| CNKI |  |
|  | SU=('heart failure' + 'cardiac insufficiency' + 'congestive heart failure') AND SU=('sarcopenia' + 'skeletal muscle reduction' + 'decreased muscle mass' + 'muscle atrophy' + 'skeletal muscle atrophy') AND SU=('diagnosis' + 'diagnostic criteria' + 'screening' + 'prevalence' + 'detection rate' + 'cutoff value' + 'cutoff' + 'diagnostic efficacy' + 'sensitivity' + 'specificity') |
| Wanfang |  |
|  | Subject/Topic: (("heart failure" OR "cardiac insufficiency" OR "congestive heart failure") AND ("sarcopenia" OR "skeletal muscle reduction" OR "decreased muscle mass") AND ("diagnosis" OR "diagnostic criteria" OR "screening" OR "prevalence" OR "detection rate" OR "cutoff value" OR "cutoff")) |

Note: Search strategies for CNKI and Wanfang are reported in English translation; the original strategies were executed in Chinese.
